# ICAT: a simple score predicting critical care needs after thrombolysis in stroke patients

**DOI:** 10.1186/s13054-016-1195-7

**Published:** 2016-01-28

**Authors:** Roland Faigle, Elisabeth B. Marsh, Rafael H. Llinas, Victor C. Urrutia, Rebecca F. Gottesman

**Affiliations:** Department of Neurology, Johns Hopkins University School of Medicine, 600 N Wolfe Street, Phipps 484, Baltimore, MD 21287 USA

**Keywords:** Critical care needs, Ischemic stroke, Thrombolysis, Thrombolytics, Risk prediction score

## Abstract

**Background:**

Patients receiving intravenous thrombolysis (IVT) for acute ischemic stroke are at risk of developing complications, commonly necessitating admission to an intensive care unit (ICU). At present, most IVT is administered in the Emergency Department or in dedicated stroke units, but no evidence-based criteria exist that allow for early identification of patients at increased risk of developing ICU needs. The present study describes a novel prediction score aiming to identify a subpopulation of post-IVT patients at high risk for critical care interventions.

**Methods:**

We retrospectively analyzed data from 301 patients undergoing IVT at our institutions during a 5-year period. Two hundred and ninety patients met inclusion criteria. The sample was randomly divided into a development and a validation cohort. Logistic regression was used to develop a risk score by weighting predictors of critical care needs based on strength of association.

**Results:**

Seventy-two patients (24.8 %) required critical care interventions. Black race (odds ratio [OR] 3.81, p =0.006), male sex (OR 3.79, p =0.008), systolic blood pressure (SBP; OR 1.45 per 10 mm Hg increase in SBP, p <0.001), and NIH stroke scale (NIHSS; OR 1.09 per 1 point increase in NIHSS, p =0.071) were independent predictors of critical care needs. The optimal model for score development, predicting critical care needs, achieved an AUC of 0.782 in the validation group. The score was named the ICAT (Intensive Care After Thrombolysis) score, assigning the following points: black race (1 point), male sex (1 point), SBP (2 points if 160–200 mm Hg; 4 points if >200 mm Hg), and NIHSS (1 point if 7–12; 2 points if >12). Each 1-point increase in the score was associated with 2.22-fold increased odds for critical care needs (95 % CI 1.78–2.76, p <0.001). A score ≥2 was associated with over 13 times higher odds of critical care needs compared to a score <2 (OR 13.60, 95 % CI 3.23–57.19), predicting critical care with 97.2 % sensitivity and 28.0 % specificity.

**Conclusion:**

The ICAT score, combining information about race, sex, SBP, and NIHSS, predicts critical care needs in post-IVT patients and may be helpful when triaging post-IVT patients to the appropriate monitoring environment.

**Electronic supplementary material:**

The online version of this article (doi:10.1186/s13054-016-1195-7) contains supplementary material, which is available to authorized users.

## Background

Current guidelines suggest that patients who receive intravenous thrombolysis (IVT) for acute ischemic stroke undergo resource-intensive monitoring, including frequent vital sign checks and neurological examinations, in order to allow for detection and early intervention of potential complications in the first 24 hours after IVT [1, 2]. While all patients undergo intense monitoring, typically in the setting of an intensive care unit (ICU) or dedicated stroke unit, only a subset of patients requires critical care interventions [3]. Differences in regional infrastructure, local practice, and resource availability largely drive whether post-IVT patients are admitted to an ICU or receive care in a dedicated stroke unit, and stroke units vary significantly in their capacity to provide critical care interventions [4]. As a result, most patients admitted to the ICU never require critical care resources, while others, initially triaged to a stroke unit capable of frequent vital sign checks and neurological exams but not critical care interventions, may require subsequent transfer to the ICU if complications arise. Unnecessary ICU admissions may lead to Emergency Department (ED) overcrowding and prolonged ED boarding times [5]; in addition, patients unnecessarily subjected to a critical care environment may be at increased risk of health-care associated infections and delirium associated with poor outcomes [6–8]. Conversely, delay of ICU transfer for patients in genuine need of critical care may result in poor outcome and increased mortality [9–11]. No established parameters exist that would allow for risk stratification of post-IVT patients by critical care needs, and there is currently no known scoring system that reliably identifies post-IVT patients in need of critical care or allows for identification of patients for which ICU care may be unnecessary and potentially harmful.

The purpose of the present study is to develop a clinically feasible risk prediction score to assist physicians in identifying a subpopulation of post-IVT patients in whom a high-intensity level of care may not provide additional benefit, and conversely identify patients at high risk for needing ICU care. To our knowledge, this is the first study to explore a clinical prediction score for ICU care in post-IVT patients.

## Methods

### Patients and study design

This study was approved by the Johns Hopkins University School of Medicine Institutional Review Board (IRB). Data were obtained from prospectively collected de-identified databases of patients treated for stroke at The Johns Hopkins Hospital and Johns Hopkins Bayview Medical Center between January 2010 and December 2014. A waiver of consent was granted based on paragraph 116 of the US Code of Federal Regulations, Title 45, Part 46 (45 CFR 46.116). An IRB waiver of Health Insurance Portability and Accountability Act (HIPAA) Privacy Authorization was also granted to allow review of medical records to abstract data to de-identify for use in research.

Demographic data including age, sex, and race were collected for all patients. Other variables of interest obtained from the medical record included stroke risk factors: hypertension, hyperlipidemia, diabetes mellitus, smoking status, history of atrial fibrillation, history of coronary artery disease (CAD), prior history of stroke, and the pre-hospital use of antiplatelet agents, anticoagulation, and statins. The National Institutes of Health Stroke Scale (NIHSS) and the following physiologic parameters at presentation were recorded: blood pressure (BP), serum glucose, serum creatinine, and glomerular filtration rate (GFR). Stroke location (supratentorial vs infratentorial), and laterality in supratentorial strokes (left vs right) was recorded. For patients without imaging confirmation of stroke location, the most likely localization was determined based on the clinical presentation. Data on total length of stay (LOS), length of ICU stay, discharge destination, and in-hospital mortality were collected.

A critical care intervention was considered any therapy or intervention that required ICU resources as defined previously [3]. Specifically, ICU admission criteria included: uncontrolled hypertension requiring active titration of continuous infusion of intravenous (IV) antihypertensive drugs for labile BP, use of vasopressors either for symptomatic systemic hypotension or blood pressure augmentation, need for invasive hemodynamic monitoring, uncontrolled hyperglycemia requiring IV insulin, respiratory compromise resulting in either initiation of continuous bilevel positive airway pressure (BiPAP) or mechanical ventilation, anaphylaxis, arterial bleeding requiring transfusion of blood products, management of cerebral edema and increased intracranial pressure, neurosurgical intervention such as decompressive craniectomy, or symptomatic intracerebral hemorrhage defined as any ICH with neurological deterioration, as indicated by a change in NIHSS ≥4 compared to the baseline as described previously [12].

### IV thrombolysis protocol

At our institutions, IVT is administered according to the American Heart Association’s national guidelines [1]. Post-IVT monitoring conforms to the recommendations of the Brain Attack Coalition, which have become the standard of care for most stroke centers [13]. All patients receiving IVT are monitored in the neurointensive care unit for at least 24 hours after initiation of thrombolysis, and undergo neuroimaging with either computed tomography (CT) or magnetic resonance imaging (MRI) within 24 hours after treatment before being considered for transfer to the floor.

### Statistical analysis

Statistical analysis was performed using STATA version 13 (Stata Statistical Software: Release 13. College Station, TX, USA). A *p* value <0.05 was considered statistically significant; 95 % confidence intervals are reported. For univariate analyses, continuous variables were analyzed using Student’s *t* test for normally distributed variables, and Wilcoxon rank-sum tests (Mann–Whitney U test) for non-normally distributed variables. Categorical variables were analyzed using Pearson’s Chi2 analysis, and Fisher’s exact tests, when appropriate.

The prediction model was developed using a random sample of 50 % of the dataset (development group), and was subsequently tested upon the remaining 50 % (validation group). In addition, the score was tested on the entire population after score development. A multivariable statistical model of predictors of critical care interventions was developed using basic demographic variables including age, sex, and race, and variables previously identified to predict critical care needs, such as SBP and NIHSS [3]. In addition, statistically significant variables from the simple logistic regression analyses were considered. In multivariable analysis, independent predictors significant at *p* <0.1 were included as score variables in the final score. Continuous variables significantly associated with outcome were transformed into categorical variables based on clinically and statistically meaningful subdivisions to facilitate their application in a practical score. For prediction models we used Akaike information criterion (AIC) for model selection. The discriminative ability of the respective models was determined by area under the receiver operating characteristics (ROC) curve analysis. Model calibration was assessed with the Hosmer Lemeshow test to determine goodness of fit. To generate the risk score, we assigned points to each variable proportional to its regression coefficients, rounded to the nearest integer. After testing the score in the validation sample, we performed sensitivity analysis by testing our score in our study population after omitting IV drips for BP control as an indication for ICU care.

## Results

### Patient characteristics

A total of 301 patients received IVT for presumed ischemic stroke in the EDs at The Johns Hopkins Hospital and Johns Hopkins Bayview Medical Center between January 2010 and December 2014. There were 11 patients who underwent endovascular therapy after IVT and were excluded, leaving 290 patients for further analysis.

The median age was 64 years (IQR 53–78 years); 50.3 % were male; and 47.2 % were black. The median NIHSS at presentation was 7 (IQR 5–12), and the median SBP was 159 mm Hg (IQR 142–181 mm Hg). There were 235 patients (81.0 %) who had history of hypertension, 146 (50.3 %) had history of hyperlipidemia, 78 (26.9 %) had history of diabetes mellitus, 58 (20.0 %) had history of atrial fibrillation, and 80 (27.6) had history of prior stroke or transient ischemic attack. The median hospital length of stay was 4 days (IQR 3–7 days), and the median length of ICU stay was 2 days (IQR 1–2 days). Seventeen (5.7 %) patients died during the course of their hospital stay. Further baseline patient characteristics are presented in Table 1.

**Table 1:** Baseline characteristics of all IVT patients by critical care needs (n = 290)

Characteristics	All patients	With critical care needs	Without critical care needs	*p* value
	(n = 290)	(n = 72)	(n = 218)	
Age, years: median (IQR)	64 (53–78)	68.5 (57–79.5)	63 (51–77)	0.038
Sex, male: n (%)	146 (50.3)	42 (58.3)	104 (47.7)	0.118
Race, black: n (%)	137 (47.2)	45 (62.5)	92 (42.2)	0.003
NIHSS: median (IQR)	7 (5–12)	9 (5–16)	7 (5–11)	<0.001
BP, mm Hg: median (IQR)				
SBP	159 (142–181)	180 (161–203)	152 (140–174)	<0.001
DBP	90 (80–100)	100 (87–112)	87 (79–100)	<0.001
IVT window <3 h: n (%)	202 (69.7)	48 (66.7)	154 (70.6)	0.525
Medical comorbidities: n (%)				
Hypertension	235 (81.0)	65 (90.3)	170 (78.0)	0.021
Hyperlipidemia	146 (50.3)	38 (52.8)	108 (49.5)	0.634
Diabetes mellitus	78 (26.9)	23 (31.9)	55 (25.2)	0.265
Coronary artery disease	69 (23.8)	17 (23.6)	52 (23.9)	0.967
Reduced ejection fraction	28 (9.7)	8 (11.1)	20 (9.2)	0.639
Atrial fibrillation	58 (20.0)	19 (26.4)	39 (17.9)	0.118
Prior ischemic stroke/TIA	80 (27.6)	22 (30.6)	58 (26.6)	0.516
Smoking	92 (31.8)	18 (25.0)	74 (33.9)	0.157
Medications: n (%)				
Antiplatelet agent	130 (44.8)	31 (43.1)	99 (45.4)	0.727
Anticoagulation	17 (5.9)	4 (5.6)	13 (6.0)	1.000
Statin	110 (37.9)	23 (31.9)	87 (39.9)	0.227
Glucose, mg/dl: median (IQR)	120 (103–157)	129 (106–166)	117 (101–148)	0.036
Creatinine, mg/dl: median (IQR)	1.0 (0.9-1.3)	1.1 (0.9-1.4)	1.0 (0.8-1.3)	0.008
GFR <60 ml/min: n (%)	87 (30.0)	23 (31.9)	64 (29.4)	0.678
Stroke location^a^: n (%)				0.204
Supratentorial	268 (92.4)	64 (88.9)	204 (93.6)	
Right-hemispheric	119 (44.4)	31 (48.4)	88 (43.1)	
Left-hemispheric	149 (55.6)	33 (51.6)	116 (56.9)	
Infratentorial	22 (7.6)	8 (11.1)	14 (6.4)	
LOS, days: median (IQR)	4 (3–7)	6 (5–12.5)	4 (3–6)	<0.001
ICU stay, days: median (IQR)	1 (1–2)	2 (2–5)	1 (1–2)	<0.001
Final diagnosis: n (%)				0.074
Stroke	247 (85.2)	66 (91.7)	181 (83.0)	
Stroke mimic	43 (14.8)	6 (8.3)	37 (17.0)	
Discharge to home: n (%)	163 (56.2)	21 (29.2)	142 (65.1)	<0.001
Mortality: n (%)	17 (5.9)	15 (20.8)	2 (0.9)	<0.001

^a^Location was confirmed by neuroimaging, or was presumed based on clinical presentation in cases where subsequent imaging was negative for ischemia; *p* values are for comparison of patients with and without critical care needs. *BP* blood pressure, *DBP* diastolic BP, *GFR* glomerular filtration rate, *ICU* intensive care unit, *IQR* interquartile range, *IVT* intravenous thrombolysis, *LOS* length of stay, *NIHSS* NIH Stroke Scale, *SBP* systolic BP, *TIA* transient ischemic attack

Seventy-two patients (24.8 %) underwent critical care interventions during their hospitalization. The characteristics of patients with and without need for critical care were compared (Table 1). Patients who required critical care were older (median age 68.5 vs 63 years), more likely to be black (62.5 % vs 42.2 %), had higher NIHSS at presentation (median 9 vs 7), higher SBP (median 180 vs 152 mm Hg), and were more likely to have a history of hypertension (90.3 % vs 78.0 %). The critical care group was less likely to be discharged to home (29.2 % vs 65.1 %), and had higher mortality rates (20.8 % vs 0.9 %).

Among the 72 patients who underwent ICU care, 23 (31.9 %) required two or more critical care interventions. The most common ICU interventions comprised titration of continuous infusions of IV antihypertensive drugs for uncontrolled hypertension (37/72 patients; 51.4 %), respiratory and airway compromise (27/72; 37.5 %), and management of cerebral edema (15/72; 20.8 %). A complete list of critical care interventions can be found in Additional file 1: Table S1.

### Development of the critical care prediction model

Simple logistic regression in the development group identified the following clinical and physiologic characteristics associated with need for critical care: black race (odds ratio (OR) 2.67, *p* = 0.015), male sex (OR 2.39, *p* = 0.032), SBP (OR 1.38 per 10 mm Hg increase in SBP, *p* <0.001), diastolic blood pressure (DBP) (OR 1.42 per 10 mm Hg increase in DBP, *p* <0.001), history of hypertension (OR 12.17, *p* = 0.016), and serum glucose level (OR 1.39 per 50 mg/dl increase in serum glucose, *p* = 0.031). In multivariable logistic regression only black race, male sex, SBP, and NIHSS were independent predictors of critical care needs with *p* <0.1 (Table 2); DBP and history of hypertension were not included because of collinearity with SBP.

**Table 2:** Multivariable analysis for predictors of critical care needs in the development group

Variable	Odds ratio	95 % CI	*p* value
Age, per 10 years	1.33	0.94-1.89	0.102
Black race	3.81	1.46-9.93	0.006
Male sex	3.79	1.42-10.10	0.008
Systolic blood pressure, per 10 mm Hg	1.45	1.19-1.77	<0.001
Glucose, per 50 mg/dl	1.25	0.89-1.77	0.193
National Institutes of Health Stroke Scale	1.09	0.99-1.19	0.071

For score development, the best model included race, sex, SBP with cut points at 160 mm Hg and 200 mm Hg, and NIHSS with cut points at 7 and 13. This model produced an AUC of 0.861 (95 % CI 0.781, 0.940) and the Hosmer Lemeshow test confirmed goodness of fit (*p* = 0.165).

### Model validation and risk score

**Fig. 1:**
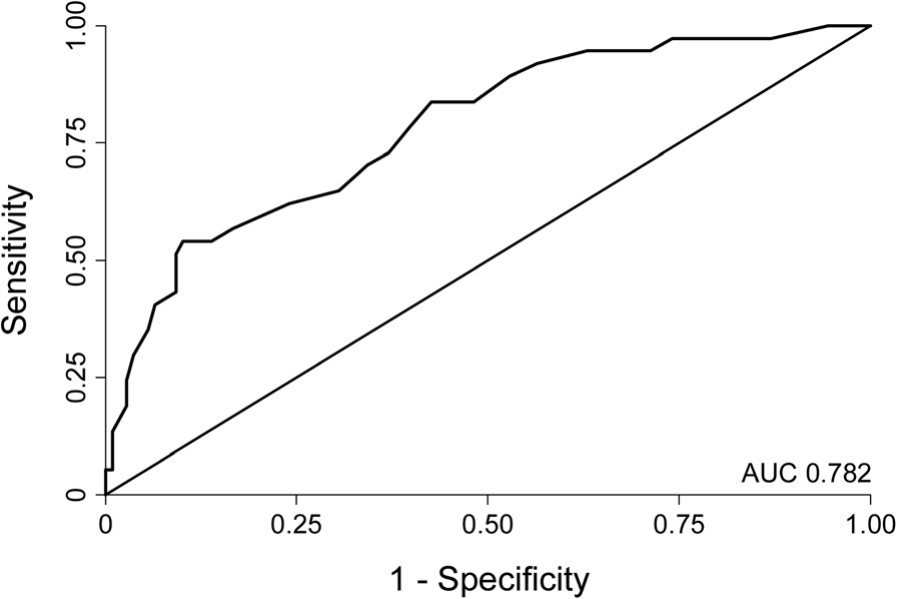
Receiver operating characteristics curve for the score model predicting critical care needs in the validation group. The area under the curve (*AUC*) of 0.782 shows that the model is predictive of critical care after intravenous thrombolysis

In the validation group, the AUC for the complete model was 0.782 (95 % CI 0.696, 0.868; Fig. 1), and the model fit the data well (Hosmer Lemeshow, *p* = 0.638). A four-item risk score, the Intensive Care After Thrombolysis (ICAT) score, was developed based on the following model:

$$ \mathrm{Log}\left(\mathrm{odds}\right)\mathrm{I}\mathrm{C}\mathrm{U} = -4.38+1.01{\mathrm{x}}_1+1.29{\mathrm{x}}_2+2.11{\mathrm{x}}_3+4.11{\mathrm{x}}_4+0.75{\mathrm{x}}_5+1.61{\mathrm{x}}_6 $$

where x_1_ = 1 if race = black, x_2_ = 1 if sex = male, x_3_ = 1 if SBP 160–200 mm Hg, x_4_ = 1 if SBP >200, x_5_ = 1 NIHSS 7–12, x_6_ = 1 if NIHSS >12. Thus, based on the regression coefficients, points for the ICAT score were assigned as follows: 1 point for black race; 1 point for male sex; 2 points for SBP 160–200 mm Hg, 4 points for SBP >200 mm Hg; 1 point for NIHSS 7–12, and 2 points for NIHSS >12, with a maximum of 8 points (Table 3). The model for the final score produced an AUC of 0.810 (95 % CI 0.752, 0.868) in the entire sample. Each 1-point increase in the score was associated with a 2.22-fold increase in odds for critical care needs (95 % CI 1.78, 2.76, *p* <0.001). The rates for critical care needs in patients with a score of 0–1, 2–4, and 5–8 were 2/63 (3.2 %), 37/181 (20.4 %), and 33/46 (71.7 %), respectively. The odds of requiring critical care for a patient with a score ≥2 was over 13 times higher than for a patient with a score <2 (OR 13.60, 95 % CI 3.23, 57.19). This cut point predicted critical care needs with 97.2 % sensitivity and 28.0 % specificity (Table 4). A score ≥5 predicted need for ICU care with 45.8 % sensitivity and 94.0 % specificity (OR 13.34, 95 % CI 6.44, 27.62). Table 4 shows the combination of sensitivity and specificity for each clinically relevant score cut point.

**Table 3:** Determination of the Intensive Care After Thrombolysis (ICAT) score

Score component	ICAT score points
Male sex	
No	0
Yes	1
Black race	
No	0
Yes	1
Systolic blood pressure, mm Hg	
<160	0
160-200	2
>200	4
National Institutes of Health Stroke Scale, score	
≤6	0
7–12	1
≥13	2
Total score	0–8

**Table 4:** Sensitivity and specificity of the Intensive Care After Thrombolysis (ICAT) score by cut point

ICAT cut point	Sensitivity (%)	Specificity (%)	Correctly classified (%)
≥2	97.2	28.0	45.2
≥3	84.7	55.1	62.4
≥4	69.4	80.7	77.9
≥5	45.8	94.0	82.1
≥6	23.6	97.3	79.0

As some stroke units have the capability of IV drips for blood pressure control, we performed a sensitivity analysis applying our score to predict need for ICU care, however, redefining the outcome by discounting IV drips for blood pressure control as a need for ICU care. Even under a paradigm that does not consider IV drips for blood pressure control a requirement for an ICU stay, each 1-point increase in the score was associated with a 1.69-fold increase in odds for critical care needs (95 % CI 1.38, 2.08, *p* <0.001), and our score achieved an AUC of 0.753 (95 % CI 0.675, 0.831).

## Discussion

In the present study we identified risk factors for critical care interventions in patients with acute stroke, undergoing IVT. We derived and validated a simple risk score based on patient race, sex, stroke severity identified by the NIHSS, and SBP. All components of the score are easily obtainable, and are part of routine clinical practice.

We have previously shown that black patients have higher risk of requiring critical care interventions post-IVT compared to white patients [3]. Black patients and patients from other minorities are well-documented to have higher health care utilization at the end of life due to lower rates of withdrawal of care [14–17]. Although withdrawal of care status was not formally collected in our study, the overall mortality rate was low in our study population and did not differ by race (5.8 % in black vs 5.9 % in white patients); therefore, differences in aggressiveness of care by race is unlikely to explain the observed differences in ICU care. In addition, most critically ill patients in our study required ICU care within the first 24 hours of presentation, thus, sooner than the typical time period of discussion on care withdrawal. Black patients have higher rates of malignant hypertension compared to white patients. However, the observed racial disparities for critical care in our population persisted after excluding patients whose sole ICU indication was IV antihypertensive medication for uncontrolled hypertension.

Men had higher odds of critical care interventions compared to women. This finding is consistent with other studies suggesting that women overall receive less aggressive care after stroke compared to their male counterparts [18–20]. In addition, our finding is in line with other studies suggesting that male sex is a risk factor for admission to general ICUs [21] and unplanned ICU readmissions [22], and that men in general are more likely to receive life-sustaining interventions compared to women [23, 24]. Differences in stroke severity or severity of other measured comorbidities are unlikely to explain the observed sex and race disparities as 1) they are accounted and adjusted for in our regression model, and 2) they do not differ substantially between black vs white (Additional file 2: Table S2) or male vs female (Additional file 3: Table S3) patients, respectively. Reasons for race and sex differences in health care are multifactorial and insufficiently understood. It is therefore likely that other variables not captured in our study contribute to the observed differences by sex and race, such as bias in provider decision-making or provider-patient/decision-maker interaction [25–27].

Determining the optimal cut point for clinical use of any score is highly dependent on the risks and consequences of false-positive and false-negative predictions. When triaging patients post-IVT, safety and the ability to rapidly detect and respond to complications is of utmost importance, thus favoring a cut point with high sensitivity and minimal rate of false-negatives (patients falsely predicted as not requiring ICU care who later end up needing ICU care). For the practical application of our score we propose a cut point of 2, which was associated with >97 % sensitivity in identifying patients in need of critical care interventions; however, some clinicians may prefer to choose a higher cut point rendering higher specificity at the expense of sensitivity, for their own practice.

While it is recognized that some but not all patients require intensive monitoring post-IVT, there are currently no evidence-based guidelines that would allow for appropriate risk stratification of post-IVT patients, and the monitoring environment for post-IVT patients is highly variable across the world. Currently, admission of post-IVT patients to an ICU vs stroke unit (if available) is largely dependent on the individual clinical judgment of the admitting physician, applying criteria commonly employed for ICU triaging in general, such as perceived vital sign instability, laboratory abnormalities, airway compromise, or mental status compromise. However, some complications may not be present at the time of triaging; thus, our score may be helpful in identifying patients with high likelihood of future need of critical care and may prompt a priori triaging of post-IVT patients to the appropriate monitoring environment. Our score does not specifically predict hemorrhagic transformation, but rather the composite of all complications resulting in need for ICU care. While in the USA IVT is typically administered in the ED, most European centers and hospitals directly admit the patient to an ICU or stroke unit where IVT is administered. As our score can be computed in the pre-hospital stage, it may aid the initial triaging of the patient to the appropriate monitoring environment, and thereby reduce subsequent patient transfers and hand-off among physician teams.

Our study has several limitations. Limiting the number of score variables to allow for easy score computation comes at the expense of accuracy of outcome prediction. Other neuroimaging characteristics may be associated with critical care needs after IVT, such as quantitative infarct volume [28]; however, we did not include this variable in our model for score development in order to allow for easy score computation prior to IVT without need for additional non-routine imaging. Our score is intended to be applicable for all stroke subtypes. However, as the vast majority of patients included had strokes originating from a supratentorial location, our score is best generalizable to IVT patients presenting with presumed ischemia in supratentorial locations. We acknowledge that the validity of our score for some infratentorial locations, such as large cerebellar stroke, is uncertain because our study was not adequately powered to validate score performance in less common stroke locations. Thus, applying our score to patients with presumed cerebellar stroke must be done with caution. Indications for ICU admission and interventions may differ among institutions across the US and worldwide, and the model described in this study might therefore not be valid in institutions where ICU admission criteria differ significantly from ours. Although we validated our score in a separate cohort, the patients in the validation cohort were cared for by the same physicians, in the same infrastructure setting, and over the same time period as the patients in the development cohort. Thus, it is possible that results may vary in an entirely different patient population cared for by different physicians in a different infrastructure setting. As we excluded patients undergoing endovascular therapy, our results are only generalizable to patients receiving IVT without subsequent endovascular therapy. However, despite recent proof of efficacy of endovascular therapy for some patients undergoing IVT [29], we anticipate that our score will still be applicable to the vast majority of IVT cases, as only a relatively small subset of post-IVT patients is eligible for clot retrieval. Black race was a risk factor for ICU care in our biracial study population; however, future studies are required to determine whether this finding is specific to African Americans or applicable to other minorities in the USA or elsewhere. Last, this is a retrospective analysis of a small number of patients from two single stroke centers over the course of 5 years, limiting generalizability to larger populations. Further validation of our score in an external dataset is required.

In summary, the ICAT score incorporates information on patient race, sex, NIHSS, and SBP on admission to reliably predict critical care needs in post-IVT patients. The ICAT score can be easily computed in the pre-hospital phase, and a score ≥2 predicts critical care needs with >97 % sensitivity. We hope that our score will aid clinicians in making improved triaging decisions in stroke patients who have undergone IVT.

## Conclusion

In the present study, we describe a novel risk prediction score identifying patients with acute stroke, who are at increased risk for critical care needs after IVT. Our score, combining information about race, sex, SBP, and NIHSS, predicts critical care needs with high sensitivity, and may be helpful when triaging post-IVT patients to the appropriate monitoring environment.

## Key messages

- Black race, male sex, systolic blood pressure, and NIH Stroke Scale are components of a new risk prediction score for critical care needs in stroke patients after IVT, for which the AUC was 0.782 in the validation group
- Each 1-point increase in the score was associated with 2.22-fold increased odds for critical care needs (95 % CI 1.78, 2.76, *p* <0.001)
- A score ≥2 was associated with over 13 times higher odds of critical care needs compared to a score <2 (OR 13.60, 95 % CI 3.23, 57.19), predicting critical care with 97.22 % sensitivity

## Additional files

Additional file 1: Table S1. Complete list of the nature of critical care interventions among patients with ICU needs (n = 72). Categories are not mutually exclusive. *BP* blood pressure, *ICU* intensive care unit, *ICH* intracerebral hemorrhage, *IV* intravenous, *ggt* drip. (DOCX 18 kb)

Additional file 2: Table S2. Baseline characteristics of all intravenous thrombolysis (*IVT*) patients stratified by race (n = 290). *Location was confirmed by neuroimaging, or was presumed based on clinical presentation in cases where subsequent imaging was negative for ischemia; *p* values are for comparison of black patients and white patients. *BP* blood pressure, *DBP* diastolic BP, *GFR* glomerular filtration rate, *ICU* intensive care unit, *IQR* interquartile range, *LOS* length of stay, *NIHSS* NIH Stroke Scale, *SBP* systolic BP, *TIA* transient ischemic attack. (DOCX 23 kb)

Additional file 3: Table S3. Baseline characteristics of all intravenous thrombolysis (*IVT*) patients stratified by sex (n = 290). *Location was confirmed by neuroimaging, or was presumed based on clinical presentation in cases where subsequent imaging was negative for ischemia; *p* values are for comparison of black patients and white patients. *BP* blood pressure, *DBP* diastolic BP, *GFR* glomerular filtration rate, *ICU* intensive care unit, *IQR* interquartile range, *LOS* length of stay, *NIHSS* NIH Stroke Scale, *SBP* systolic BP, *TIA* transient ischemic attack. (DOCX 23 kb)

## Abbreviations

AIC: Akaike information criterion
AUC: area under the curve
BiPAP: bilevel positive airway pressure
BP: blood pressure
CAD: coronary artery disease
CFR: Code of Federal Regulations
CI: confidence interval
DBP: diastolic blood pressure
ED: Emergency Department
GFR: glomerular filtration rate
HIPAA: Health Insurance Portability and Accountability Act
ICU: intensive care unit
IQR: interquartile range
IRB: Institutional Review Board
IV: intravenous
IVT: intravenous thrombolysis
LOS: length of stay
NIHSS: National Institutes of Health Stroke Scale
OR: odds ratio
ROC: receiver operating characteristics
SBP: systolic blood pressure
