# Supplementary material for: ICAT: a simple score predicting critical care needs after thrombolysis in stroke patients
Source: Crit Care. 2016 Jan 28;20:26. doi: 10.1186/s13054-016-1195-7 (PMC4730614; doi:10.1186/s13054-016-1195-7)
Supplement: Supplementary file 1 — Complete list of the nature of critical care interventions among patients with ICU needs (n = 72). Categories are not mutually exclusive. BP blood pressure, ICU intensive care unit, ICH intracerebral hemorrhage, IV intravenous, ggt drip. (DOCX 18 kb) [file 13054_2016_1195_MOESM1_ESM.docx]

**Additional file 1: Table S1. Complete list of the nature of critical care interventions among patients with ICU needs (n=72).** BP: blood pressure; ICU: intensive care unit; ICH: intracerebral hemorrhage; IV: intravenous; ggt: drip. Categories are not mutually exclusive.

| **Critical Care Intervention** | **N (%)** |
| --- | --- |
| IV antihypertensive ggt | 37 (51.4) |
| Respiratory/airway compromise | 27 (37.5) |
| Management of cerebral edema | 15 (20.8) |
| Symptomatic ICH | 8 (11.1) |
| BP augmentation | 6 (8.3) |
| IV heart rate/rhythm control ggt | 6 (8.3) |
| Other | 3 (4.2) |
| **Any critical care intervention** | **72 (100)** |
