# Supplementary material for: ICAT: a simple score predicting critical care needs after thrombolysis in stroke patients
Source: Crit Care. 2016 Jan 28;20:26. doi: 10.1186/s13054-016-1195-7 (PMC4730614; doi:10.1186/s13054-016-1195-7)
Supplement: Supplementary file 3 — Baseline characteristics of all intravenous thrombolysis (IVT) patients stratified by sex (n = 290). *Location was confirmed by neuroimaging, or was presumed based on clinical presentation in cases where subsequent imaging was negative for ischemia; p values are for comparison of black patients and white patients. BP blood pressure, DBP diastolic BP, GFR glomerular filtration rate, ICU intensive care unit, IQR interquartile range, LOS length of stay, NIHSS NIH Stroke Scale, SBP systolic BP, TIA transient ischemic attack. (DOCX 23 kb) [file 13054_2016_1195_MOESM3_ESM.docx]

**Additional file 3: Table S3. Baseline characteristics of all IVT patients stratified by sex (N=290).** BP: blood pressure; DBP: diastolic BP; GFR: glomerular filtration rate; ICU: intensive care unit; IQR: interquartile range; IVT: intravenous thrombolysis; LOS: length of stay; NIHSS: NIH Stroke Scale; SBP: systolic BP; TIA: transient ischemic attack; *Location was confirmed by neuroimaging, or was presumed based on clinical presentation in cases where subsequent imaging was negative for ischemia; P-values compare male and female patients.

| **Characteristics** | **Male**  *(n=146)* | **Female**  *(n=144)* | **p-value** |
| --- | --- | --- | --- |
| **Age** – years: median (IQR) | 63 (53-73) | 68 (53-82) | 0.029 |
| **Race** – black: n (%) | 60 (41.1) | 77 (53.5) | 0.035 |
| **NIHSS** – median (IQR) | 7 (4-11) | 8 (5-12) | 0.165 |
| **BP** – mm Hg: median (IQR) |  |  |  |
| SBP | 159 (144-180) | 160 (141-183) | 0.846 |
| DBP | 90 (80-102) | 89 (80-100) | 0.727 |
| **IVT window <3hrs** – n (%) | 106 (72.6) | 96 (66.7) | 0.272 |
| **Medical Comorbidities** – n (%) |  |  |  |
| Hypertension | 113 (77.4) | 122 (84.7) | 0.112 |
| Hyperlipidemia | 70 (48.0) | 76 (52.8) | 0.441 |
| Diabetes mellitus | 37 (25.3) | 41 (28.5) | 0.548 |
| Coronary artery disease | 46 (31.5) | 23 (16.0) | 0.002 |
| Reduced ejection fraction | 18 (12.3) | 10 (6.9) | 0.163 |
| Atrial fibrillation | 29 (19.9) | 29 (20.1) | 0.953 |
| Prior ischemic stroke/TIA | 32 (21.9) | 48 (33.3) | 0.030 |
| Smoking | 50 (34.3) | 42 (29.2) | 0.353 |
| **Medications** – n (%) |  |  |  |
| Antiplatelet agent | 61 (41.8) | 69 (47.9) | 0.293 |
| Anticoagulation | 9 (6.2) | 8 (5.6) | 1.000 |
| Statin | 53 (36.3) | 57 (39.6) | 0.565 |
| **Glucose** – mg/dl: median (IQR) | 119 (103-159) | 121 (101-150) | 0.727 |
| **Creatinine** – mg/dl: median (IQR) | 1.1 (1.0-1.3) | 0.9 (0.8-1.2) | <0.001 |
| **GFR < 60 ml/min** – n (%) | 39 (26.7) | 48 (33.3) | 0.219 |
| **Stroke location*** – n (%) |  |  | 0.044 |
| Supratentorial | 130 (89.0) | 138 (95.8) |  |
| *Right-hemispheric* | *61 (46.9)* | *58 (42.0)* |  |
| *Left-hemispheric* | *69 (53.1)* | *80 (58.0)* |  |
| Infratentorial | 16 (11.0) | 6 (4.2) |  |
| **LOS** – days: median (IQR) | 5 (3-6) | 4 (3-6) | 0.343 |
| **ICU stay –** days: median (IQR) | 2 (1-2) | 1 (1-2) | 0.128 |
| **Final diagnosis** – n (%) |  |  | 0.062 |
| Stroke | 130 (89.0) | 117 (81.3) |  |
| Stroke mimic | 16 (11.0) | 27 (18.7) |  |
| **Discharge to home** – n (%) | 84 (57.5) | 79 (54.9) | 0.871 |
| **Mortality** – n (%) | 7 (4.8) | 10 (6.9) | 0.465 |
